# Tucker Decomposition-Based Feature Selection and SSA-Optimized Multi-Kernel SVM for Transformer Fault Diagnosis

**DOI:** 10.3390/s25247547

**Published:** 2025-12-12

**Authors:** Luping Wang, Xiaolong Liu

**Affiliations:** School of Automation, Shenyang Aerospace University, Shenyang 110136, China; liuxiaolong@stu.sau.edu.cn

**Keywords:** transformer fault diagnosis, dissolved gas analysis, Tucker decomposition, feature selection, multi-kernel support vector machine

## Abstract

Accurate fault diagnosis of power transformers is critical for maintaining grid reliability, yet conventional dissolved gas analysis (DGA) methods face challenges in feature representation and high-dimensional data processing. This paper presents an intelligent diagnostic framework that synergistically integrates systematic feature engineering, tensor decomposition-based feature selection, and a sparrow search algorithm (SSA)-optimized multi-kernel support vector machine (MKSVM) for transformer fault classification. The proposed approach first expands the original five-dimensional gas concentration measurements to a twelve-dimensional feature space by incorporating domain-driven IEC 60599 ratio indicators and statistical aggregation descriptors, effectively capturing nonlinear interactions among gas components. Subsequently, a novel Tucker decomposition framework is developed to construct a three-way tensor encoding sample–feature–class relationships, where feature importance is quantified through both discriminative power and structural significance in low-rank representations, successfully reducing dimensionality from twelve to seven critical features while retaining 95% of discriminative information. The multi-kernel SVM architecture combines radial basis function, polynomial, and sigmoid kernels with optimized weights and hyperparameters configured through SSA’s hierarchical producer–scrounger search mechanism. Experimental validation on DGA samples across seven fault categories demonstrates that the proposed method achieves 98.33% classification accuracy, significantly outperforming existing methods, including kernel PCA-based approaches, deep learning models, and ensemble techniques. The framework establishes a reliable and accurate solution for transformer condition monitoring in power systems.

## 1. Introduction

Power transformers constitute critical nodes in electrical transmission and distribution networks, serving as the primary interface for voltage transformation and power transfer between grid segments [1]. The operational integrity of these assets directly governs grid reliability, with unexpected failures potentially triggering cascading outages and substantial economic losses. Statistical evidence indicates that transformer malfunctions account for approximately 40% of major power system disruptions worldwide, with repair costs often exceeding millions of dollars per incident alongside prolonged downtime periods. Given the capital-intensive nature of transformer infrastructure, developing robust fault diagnosis methodologies for early detection and accurate classification of incipient defects has emerged as essential for modern power system maintenance strategies [2]. Timely identification of developing faults enables condition-based maintenance scheduling, extends asset lifespan through proactive intervention, and enhances grid resilience against sudden component failures [3].

Dissolved gas analysis (DGA) has established itself as the predominant non-invasive diagnostic technique for oil-immersed power transformers [4], leveraging the principle that thermal and electrical stresses induce decomposition of insulating oil and cellulose materials, thereby generating characteristic dissolved gas signatures. The concentrations of key fault gases—hydrogen (H_2_), methane (CH_4_), ethane (C_2_H_6_), ethylene (C_2_H_4_), and acetylene (C_2_H_2_)—provide critical forensic evidence for differentiating among diverse fault mechanisms, including thermal overheating at various temperature ranges, partial discharge phenomena, and electrical arcing events [5]. Conventional DGA interpretation frameworks, exemplified by the IEC 60599 three-ratio method, Duval triangle, and Rogers ratio codes, employ expert-defined thresholds and geometric boundaries to partition the gas concentration space into fault categories [6]. While these rule-based approaches offer interpretability and have accumulated decades of field validation, they exhibit inherent limitations in handling borderline cases, overlapping fault signatures, and complex multi-fault scenarios. Furthermore, the rigid decision boundaries fail to adapt to evolving operational conditions, transformer designs, or region-specific oil degradation characteristics, consequently constraining diagnostic accuracy in heterogeneous deployments.

The advent of machine learning has catalyzed a paradigm shift toward data-driven transformer fault diagnosis, with support vector machines [7], artificial neural networks [8], ensemble methods, and deep learning architectures [9] demonstrating superior classification performance compared to conventional rule-based systems. Despite these advances, contemporary intelligent diagnostic frameworks confront persistent challenges across multiple dimensions. First, the inherently low-dimensional nature of raw DGA measurements—typically comprising only five gas concentrations—restricts the feature space’s representational capacity for capturing subtle fault distinctions [10]. Second, feature engineering strategies that expand the original measurement space inevitably introduce feature redundancy and multicollinearity, necessitating principled dimensionality reduction to prevent classifier overfitting [11]. Third, existing feature selection methodologies predominantly operate on matrix-based data representations, thereby overlooking the intrinsic three-way coupling among samples, features, and fault classes—a structural characteristic that could yield more discriminative feature subsets [12]. Fourth, classifier hyperparameter optimization remains computationally demanding, especially for multi-kernel architectures where the high-dimensional hyperparameter space renders exhaustive grid search prohibitively expensive [13].

Recently, the field of transformer fault diagnosis has seen the adoption of SVMs based on kernel functions and metaheuristic optimization. Dhiman et al. [14] applied PSO to a single-kernel SVM, achieving 94.1% accuracy; However, this was constrained by the inherent limitations of the single-kernel structure. Recent metaheuristic algorithms, such as the Genetic Algorithm [15], Grey Wolf Optimizer [16], and Whale Optimization Algorithm [17], have been utilized for SVM hyperparameter tuning. Nonetheless, these studies remain predominantly within the single-kernel framework and have not systematically addressed the challenges associated with multi-kernel fusion or high-dimensional feature spaces.

Existing feature engineering methods based on DGA include ratio-based feature extraction, statistical dimensionality reduction, and wrapper-based feature selection. The IEC 60599 three-ratio method and the Duval triangle provide domain-driven feature extraction, but their discriminative power is limited when used in isolation. Although PCA and KPCA are widely employed for dimensionality reduction, their matrix operations struggle to preserve the three-dimensional structural relationships among samples, features, and fault types. Among wrapper methods, strategies based on the GA and Recursive Feature Elimination incur significant computational overhead in high-dimensional scenarios. Tensor decomposition techniques have demonstrated efficacy in computer vision and signal processing, yet their application in the DGA diagnosis domain remains unexplored.

Current diagnostic frameworks face three primary challenges: First, existing MKSVMs either utilize fixed kernel weights or optimize kernel-specific hyperparameters independently, making it impossible to obtain a globally optimal configuration. Second, mainstream feature selection methods operate in “flattened” vector spaces, neglecting the three-dimensional coupling among sample distribution, feature interactions, and class discrimination patterns—structures that can be processed via tensor algebra. Third, there is a lack of systematic comparative studies on metaheuristic algorithms specifically for transformer diagnosis, and convergence analysis within high-dimensional hyperparameter spaces is limited.

Motivated by these challenges and inspired by recent advances in tensor decomposition theory and swarm intelligence optimization, this paper proposes a comprehensive diagnostic framework that synergistically integrates systematic feature engineering, Tucker decomposition-based feature selection, and a sparrow search algorithm (SSA)-optimized multi-kernel support vector machine (MKSVM) for enhanced transformer fault classification [18]. The main contributions of this work are threefold:

1. Feature selection based on tensor decomposition is proposed, which differs from traditional matrix-based dimensionality reduction methods that discard multiple types of structural information. The proposed Tucker decomposition framework explicitly encodes sample–feature–class relationships through three-way tensor construction. The importance of features is quantified through a composite metric that balances discriminative power and structural importance in low-rank representations, which is fundamentally different from the computational burden of wrapper methods and the neglect of feature class interactions in filtering methods.
2. A multi-kernel SVM architecture with adaptive kernel weight assignment is formulated, synergistically combining radial basis function (RBF) kernels for local pattern recognition, polynomial kernels for global structure modeling, and sigmoid kernels for neural-like decision boundaries. All kernel-specific hyperparameters and fusion weights are jointly optimized through SSA’s hierarchical producer–scrounger mechanism, which effectively balances exploration and exploitation in the high-dimensional hyperparameter landscape.
3. The experimental verification of DGA samples spanning seven fault categories shows that the proposed framework achieves a classification accuracy of 98.33% while balancing accuracy and recall metrics, which is significantly better than kernel principal component analysis variants, deep neural networks, ensemble classifiers, and case-based reasoning methods, with an advantage of over 2.64%.

The remainder of this paper is organized as follows. Section 2 presents the complete methodology, detailing the extended feature engineering strategy, Tucker decomposition-based feature selection algorithm, sparrow search algorithm principles, and SSA-optimized multi-kernel SVM architecture. Section 3 describes the experimental dataset characteristics, presents convergence behavior and confusion matrix visualizations, and conducts comprehensive performance comparisons against representative baseline methods. Section 4 concludes this study by summarizing key findings and outlining promising directions for future research.

## 2. Materials and Methods

### 2.1. Extended Feature Engineering

Due to the low dimensionality of the DGA dataset, which contains only five features, a feature engineering approach was adopted to expand the feature space and enhance the model’s robustness and generalization capability. For the transformer DGA fault diagnosis task, this study designed a systematic feature expansion scheme, extending the original 5-dimensional gas concentration features to a 12-dimensional comprehensive feature space.

The original features comprise concentration measurements of five key dissolved gases, H_2_ (hydrogen), CH_4_ (methane), C_2_H_4_ (ethylene), C_2_H_6_ (ethane), and C_2_H_2_ (acetylene), which remain unchanged in the extended feature matrix. Building upon this foundation, three domain-driven ratio features were constructed based on the three-ratio method from the IEC 60599 international standard: the C_2_H_2_/C_2_H_4_ ratio reflects the degree of oil decomposition and fault temperature level, the CH_4_/H_2_ ratio serves as a key discriminant indicator for distinguishing thermal faults from electrical faults, and the C_2_H_4_/C_2_H_6_ ratio indicates the severity of thermal faults. To prevent division-by-zero exceptions in numerical computation, a perturbation term of $1\times 10^{-6}$ was added to the denominator during ratio calculations.

In addition, four global statistical features were extracted through horizontal statistical aggregation of the original five-dimensional gas concentrations, including the arithmetic mean of gas concentrations to characterize the overall gas content level, standard deviation to quantify the dispersion of concentration distribution, maximum value to capture anomalous peak information, and sum to reflect the cumulative effect of gas generation. The feature expansion strategy maps the original physical measurement space to a higher-dimensional feature representation space, effectively enhancing the feature set’s expressive power for fault patterns by introducing nonlinear interaction relationships among gases and statistical aggregation information [19].

Before feature selection and model training, the twelve expanded features were standardized to ensure that each feature contributed equally to the distance metrics and to prevent features with larger numerical ranges from dominating the optimization process. This paper employs the Min–Max normalization method, which transforms the feature matrix X into the standardized range [0,1] according to the following formula:

(1) $$x_{i,j}^{\mathrm{norm}}=\frac{x_{i,j}-x_{j}^{\min}}{x_{j}^{\max}-x_{j}^{\min}}$$

where $x_{i,j}$ is the original value of feature *j* for sample *i* and $x_{j}^{\min}$ and $x_{j}^{\max}$ are the minimum and maximum values of feature *j* across all samples, respectively. This step is crucial for the stability and convergence of the subsequent SSA-optimized multi-kernel SVM.

### 2.2. Tucker Decomposition-Based Feature Selection Framework

Let $X\in R^{n\times d}$ denote the feature matrix with *n* samples and *d* features and $y\in {\left\{1,2,\ldots ,c\right\}}^{n}$ represent the class labels. Traditional feature selection methods operate on matrix representations, potentially failing to capture multi-way interactions among samples, features, and class structures. This paper proposes a Tucker decomposition-based framework that encodes these three-way relationships into a higher-order tensor structure.

Through both discriminative power and structural significance in low-rank tensor representations, feature importance is evaluated. Tucker decomposition provides a principled framework by decomposing tensors into a core tensor and mode-specific factor matrices [20], thereby preserving inherent multi-way structures unlike conventional matrix factorization methods.

We construct a three-way tensor $T\in R^{n\times d\times c}$ encoding sample–feature–class relationships. For each class k∈{1,2,…,c}, the *k*-th frontal slice is defined as follows:

(2) $$T_{ijk}=x_{ij}\cdot I\left(y_{i}=k\right),\;i=1,\ldots ,n,\;j=1,\ldots ,d,\;k=1,\ldots ,c$$

where I(·) is the indicator function. This construction embeds class-specific feature patterns into separate slices, facilitating identification of discriminative features. The tensor is normalized by its Frobenius norm for numerical stability:

(3) $$\tilde{T}=\frac{T}{{∥T∥}_{F}},\;\mathrm{where}\;{∥T∥}_{F}=\sqrt{\sum_{i=1}^{n}\sum_{j=1}^{d}\sum_{k=1}^{c}T_{ijk}^{2}}$$

Tucker decomposition factorizes $\tilde{T}$ into a core tensor $G\in R^{r_{1}\times r_{2}\times r_{3}}$ and three orthogonal factor matrices $U^{\left(1\right)}\in R^{n\times r_{1}}$, $U^{\left(2\right)}\in R^{d\times r_{2}}$, and $U^{\left(3\right)}\in R^{c\times r_{3}}$:

(4) $$\tilde{T}=G\times_{1}U^{\left(1\right)}\times_{2}U^{\left(2\right)}\times_{3}U^{\left(3\right)}$$

where $\times_{k}$ denotes the mode-*k* tensor–matrix product. The mode-*k* product of tensor $T\in R^{I_{1}\times I_{2}\times I_{3}}$ with matrix $M\in R^{J\times I_{k}}$ is

(5) $${\left(T\times_{k}M\right)}_{i_{1}\ldots i_{k-1}ji_{k+1}\ldots i_{3}}=\sum_{i_{k}=1}^{I_{k}}T_{i_{1}\ldots i_{k}\ldots i_{3}}M_{ji_{k}}$$

This paper employs the HOSVD algorithm via mode-*k* unfolding and SVD operations. Mode-*k* unfolding matricizes the tensor with mode-*k* fibers as columns, yielding $T_{\left(1\right)}\in R^{n\times dc}$, $T_{\left(2\right)}\in R^{d\times nc}$, and $T_{\left(3\right)}\in R^{c\times nd}$. For each mode k∈{1,2,3}, we compute

(6) $$T_{\left(k\right)}=U^{\left(k\right)}\Sigma^{\left(k\right)}V^{(k)⊤}$$

where $U^{\left(k\right)}$ contains left singular vectors, $\Sigma^{\left(k\right)}$ holds singular values in descending order, and rank $r_{k}=\min\left(\dim_{k},\prod_{j\neq k}\dim_{j}\right)$. The core tensor is obtained by

(7) $$G=\tilde{T}\times_{1}U^{(1)⊤}\times_{2}U^{(2)⊤}\times_{3}U^{(3)⊤}$$

The core tensor G encodes intrinsic interactions among mode subspaces, where element $G_{ijk}$ quantifies the interaction strength between the *i*-th sample basis, *j*-th feature basis, and *k*-th class basis.

Feature importance is defined based on each feature’s contribution to the multi-way tensor structure [21]. For feature *j*, the importance score $s_{j}$ aggregates weighted contributions across feature mode components:

(8) $$s_{j}=\sqrt{\sum_{k=1}^{r_{2}}{\left(U_{jk}^{\left(2\right)}\right)}^{2}\cdot {∥G_{:k:}∥}_{F}^{2}}$$

where $U_{jk}^{\left(2\right)}$ represents feature *j*’s projection onto the *k*-th principal component and $G_{:k:}\in R^{r_{1}\times r_{3}}$ denotes the *k*-th lateral slice with Frobenius norm ${∥G_{:k:}∥}_{F}^{2}=\sum_{i=1}^{r_{1}}\sum_{\ell =1}^{r_{3}}G_{ik\ell }^{2}$.

This formulation combines representational power (projection coefficients) with structural significance (core tensor norms). The squared coefficient ${\left(U_{jk}^{\left(2\right)}\right)}^{2}$ quantifies feature *j*’s contribution to the *k*-th latent component, while ${∥G_{:k:}∥}_{F}^{2}$ measures the component’s importance in sample–class interactions. This importance measure is invariant to sample and class permutations, ensuring ranking depends solely on intrinsic feature characteristics. Features exhibiting strong discriminative patterns across multiple modes receive higher scores.

Given importance scores ${\left\{s_{j}\right\}}_{j=1}^{d}$, features are ranked in descending order to select the top *m* features. Let π:{1,…,d}→{1,…,d} denote the permutation satisfying $s_{\pi (1)}\geq s_{\pi (2)}\geq \ldots \geq s_{\pi (d)}$. The selected subset is

(9) $$F_{\mathrm{sel}}=\left\{\pi \left(1\right),\pi \left(2\right),\ldots ,\pi \left(m\right)\right\}$$

To assess decomposition quality, the relative reconstruction error is calculated:

(10) $$\epsilon_{\mathrm{recon}}=\frac{{∥\tilde{T}-\hat{T}∥}_{F}}{{∥\tilde{T}∥}_{F}}$$

where $\hat{T}=G\times_{1}U^{\left(1\right)}\times_{2}U^{\left(2\right)}\times_{3}U^{\left(3\right)}$ is the reconstructed tensor. Small errors validate that low-rank decomposition captures the essential structure, supporting feature importance reliability.

**Figure 1:**
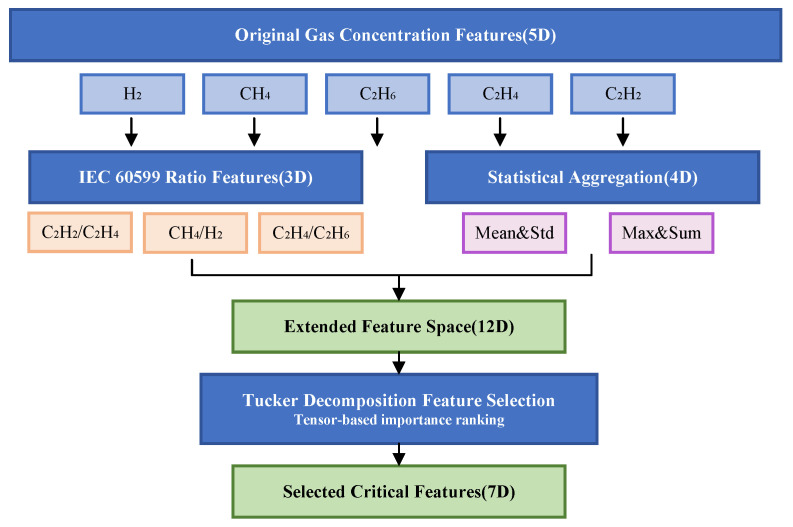
Feature extraction and selection workflow.

Additionally, the feature mode variance explained is

(11) $$\rho_{\mathrm{var}}=\frac{\sum_{k=1}^{r_{2}}{\left(\sigma_{k}^{\left(2\right)}\right)}^{2}}{\sum_{k=1}^{d}{\left(\sigma_{k}^{\left(2\right)}\right)}^{2}}\times 100\%$$

where $\sigma_{k}^{\left(2\right)}$ is the *k*-th singular value from mode-2 unfolding. This quantifies the variance retained by truncated decomposition, indicating information preservation. High ratios justify feature importance scores derived from the decomposition. Figure 1 illustrates the feature extraction and selection workflow.

### 2.3. Sparrow Search Algorithm

The sparrow search algorithm (SSA), proposed by Xue et al. in 2020 [22], simulates sparrow foraging and anti-predation behaviors. The SSA outperforms PSO and the GA on multiple benchmarks and has been widely applied in parameter optimization and feature selection [23].

The population comprises producers and scroungers. This hierarchical structure balances exploration and exploitation.

Let $X={\left[x_{1},x_{2},\ldots ,x_{n}\right]}^{T}$ denote the population position matrix, where *n* is population size, $x_{i}=\left[x_{i,1},x_{i,2},\ldots ,x_{i,d}\right]$ represents the *i*-th position in *d*-dimensional space, and $F={\left[f_{1},f_{2},\ldots ,f_{n}\right]}^{T}$ stores fitness values. The objective is $x^{*}=\arg\min_{x_{i}}f\left(x_{i}\right)$ within [lb,ub].

Producer positions update as

(12) $$x_{i}^{t+1}=\left\{\begin{array}{cc} x_{i}^{t}\cdot \exp\left(-\frac{i}{\alpha \cdot T_{\max}}\right), & \mathrm{if}\;R_{2}<ST\\ x_{i}^{t}+Q\cdot L, & \mathrm{if}\;R_{2}\geq ST \end{array}\right.$$

where $T_{\max}$ is the maximum iterations, α∈(0,1] is step size, $R_{2}\in \left[0,1\right]$ is a random variable, ST∈[0.5,1] is the safety threshold, Q is the standard normal vector, and L is a 1×d ones vector. When $R_{2}<ST$, exponential decay enables convergence; otherwise, random perturbations facilitate exploration.

Scrounger positions update as

(13) $$x_{i}^{t+1}=\left\{\begin{array}{cc} Q\cdot \exp\left(\frac{x_{\mathrm{worst}}^{t}-x_{i}^{t}}{i^{2}}\right), & \mathrm{if}\;i>n/2\\ x_{\mathrm{best}}^{t}+\left|x_{i}^{t}-x_{\mathrm{best}}^{t}\right|\cdot A^{+}\cdot L, & \mathrm{otherwise} \end{array}\right.$$

where $x_{\mathrm{best}}^{t}$ and $x_{\mathrm{worst}}^{t}$ are the best and worst positions and $A^{+}$ is the Moore–Penrose pseudoinverse of $A=A^{T}{\left(AA^{T}\right)}^{-1}$, with A being a random ±1 matrix. Better scroungers (i≤n/2) exploit the near-best position; worse ones (i>n/2) explore alternative regions.

**Figure 2:**
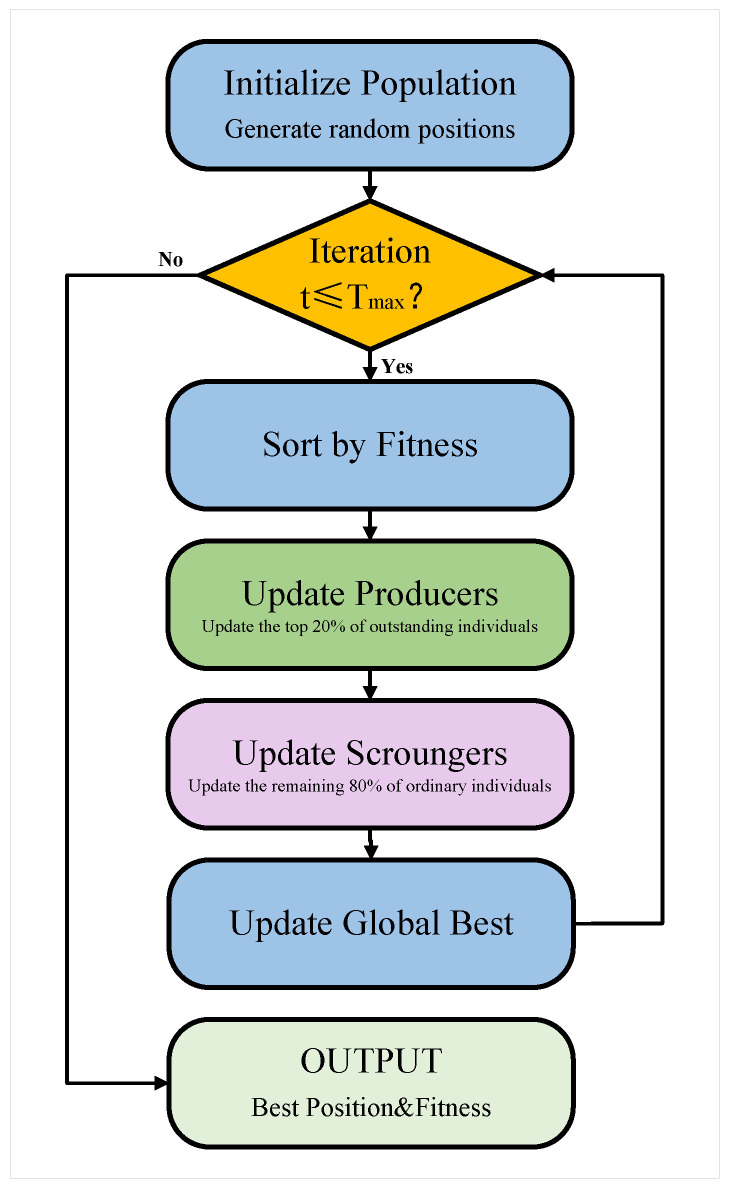
SSA algorithm flow chart.

The iterative process continues until the maximum iterations $T_{\max}$ are reached or fitness improvement falls below a threshold. Boundary constraints are enforced by truncating violations to maintain feasibility. The hierarchical structure and adaptive behavioral switching enable the SSA to achieve robust global search and fine-grained local refinement, making it effective for hyperparameter optimization in multi-kernel support vector machines [24]. The SSA algorithm flow chart is shown in Figure 2.

### 2.4. SSA-Optimized Multi-Kernel Support Vector Machine

To enhance fault discrimination capability, this paper proposes an adaptive multi-kernel SVM framework optimized by the SSA.

The sparrow search algorithm (SSA) was selected as the optimization algorithm due to its distinctive hierarchical ‘producer–scrounger’ mechanism. This mechanism provides a superior balance between global exploration and local exploitation compared to traditional metaheuristic algorithms. To validate this choice, a preliminary comparative experiment was conducted, testing four representative optimization algorithms: Particle Swarm Optimization (PSO), Genetic Algorithm (GA), Differential Evolution (DE), and SSA—all within the same multi-kernel support vector machine (MKSVM) framework. Table 1 summarizes the optimization performance across 10 independent runs, where the SSA achieved the highest average cross-validation accuracy (98.15%), the fastest convergence speed, and the smallest standard deviation of 0.12%, demonstrating its superior stability and efficiency. Compared to the population-wide exploration of PSO or the genetic operations of the GA, the SSA’s hierarchical search strategy proved more effective for navigating the ultra-high-dimensional parameter space, showing particular efficacy when addressing optimization problems involving a mixture of continuous and discrete parameters.

The composite kernel combines three base kernels to capture diverse data geometries:

(14) $$K_{\mathrm{multi}}\left(x_{i},x_{j}\right)=\sum_{k=1}^{3}w_{k}K_{k}\left(x_{i},x_{j}\right)$$

where $w_{k}\geq 0$ with $\sum_{k=1}^{3}w_{k}=1$ ensures a valid Mercer kernel. The base kernels are as follows: an RBF kernel $K_{\mathrm{RBF}}\left(x_{i},x_{j}\right)=\exp(-g_{1}∥x_{i}-x_{j}{∥}^{2})$ for local patterns, polynomial kernel $K_{\mathrm{Poly}}\left(x_{i},x_{j}\right)={\left(x_{i}^{T}x_{j}+1\right)}^{d_{2}}$ for global structures, and sigmoid kernel $K_{\mathrm{Sigmoid}}\left(x_{i},x_{j}\right)=\tanh\left(g_{3}x_{i}^{T}x_{j}+r\right)$ for neural-like boundaries.

For multi-class classification with *C* fault types, an error-correcting output code (ECOC) scheme with a one-versus-all strategy is employed. Each binary SVM solves

(15) $$\begin{array}{cc} \min_{w_{k},b_{k},\xi_{k}}\; & \frac{1}{2}{∥w_{k}∥}^{2}+c_{k}\sum_{i=1}^{n}\xi_{k,i}\\ s.t.\; & y_{i}\left(w_{k}^{T}\phi_{k}\left(x_{i}\right)+b_{k}\right)\geq 1-\xi_{k,i},\;\xi_{k,i}\geq 0 \end{array}$$

where $c_{k}$ balances margin maximization and error minimization and $\phi_{k}\left(\cdot \right)$ denotes the implicit mapping induced by kernel $K_{k}$. The decision function is

(16) $$f_{k}\left(x\right)=\mathrm{sign}\left(\sum_{i=1}^{n}\alpha_{i}^{\left(k\right)}y_{i}K_{k}\left(x_{i},x\right)+b_{k}\right)$$

Multi-kernel prediction aggregates base SVM scores via weighted fusion. For test sample x, each base model produces the score vector $s_{k}\left(x\right)\in R^{C}$. The composite score is

(17) $$s_{\mathrm{multi}}\left(x\right)=\sum_{k=1}^{3}w_{k}s_{k}\left(x\right)$$

with the predicted label

(18) $$\hat{y}=\arg\max_{j\in \{1,\ldots ,C\}}{\left[s_{\mathrm{multi}}\left(x\right)\right]}_{j}$$

The hyperparameter vector $x={\left[w_{1},w_{2},w_{3},c_{1},g_{1},c_{2},d_{2},c_{3},g_{3}\right]}^{T}\in R^{9}$ comprises kernel weights and kernel-specific parameters, bounded by [lb,ub]. The optimization problem can be formulated as follows:

(19) $$x^{*}=\arg\min_{x\in [\mathrm{lb},\mathrm{ub}]}F\left(x\right)$$

where the fitness function maximizes *k*-fold cross-validation accuracy:

(20) $$F\left(x\right)=-{\mathrm{Acc}}_{\mathrm{CV}}\left(x\right)=-\frac{1}{k}\sum_{j=1}^{k}\frac{N_{\mathrm{correct}}^{\left(j\right)}}{N_{\mathrm{total}}^{\left(j\right)}}\times 100\%$$

The SSA efficiently searches the optimal hyperparameter configuration through its hierarchical population structure. Each candidate solution is evaluated by training three base SVM models with corresponding parameters and computing their weighted cross-validation accuracy [25]. The iterative optimization process balances global exploration and local exploitation via producer–scrounger dynamics described in Section 3.2.

**Figure 3:**
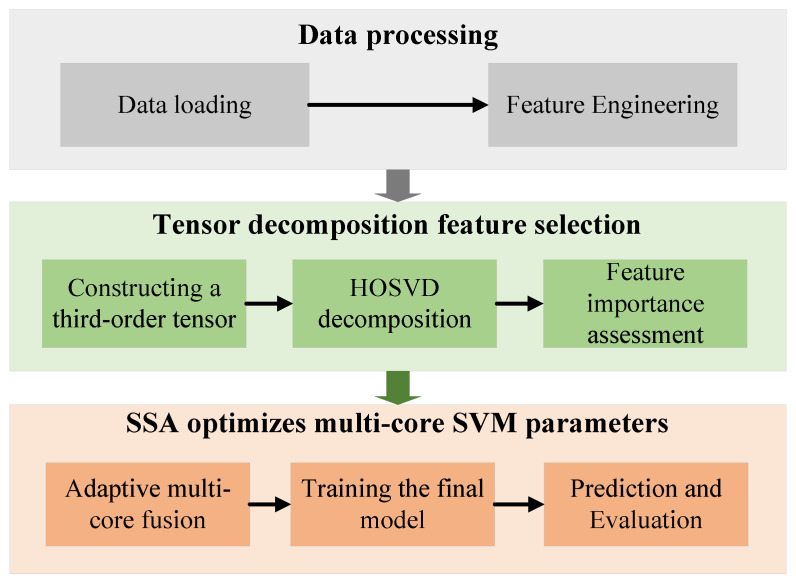
Overall flow chart.

The complete framework proceeds as follows: (1) initialize SSA population within bounds [lb,ub] with population size *M* and maximum iterations $T_{\max}$; (2) evaluate each candidate via *k*-fold cross-validation, training RBF, polynomial, and sigmoid kernel SVMs with parameters $\left(c_{1},g_{1}\right)$, $\left(c_{2},d_{2}\right)$, and $\left(c_{3},g_{3}\right)$, respectively, and then computing weighted accuracy using kernel weights $\left(w_{1},w_{2},w_{3}\right)$; (3) evolve the population through SSA position updates until convergence; (4) extract optimal hyperparameters $x^{*}$, normalize kernel weights to satisfy $\sum w_{k}=1$, and train the final multi-kernel SVM on the entire training set; and (5) classify the test samples via weighted score fusion. The overall flow chart is shown in Figure 3.

## 3. Results

### 3.1. Dataset Presentation

To ensure that the sample can represent various operating conditions, the load distribution strategy allocated approximately 25% of samples at light load levels of 30–45% rated capacity, 50% at medium load levels of 45–70% rated capacity, and 25% at heavy load levels of 70–85% rated capacity. For each fault type, samples were proportionally distributed across load levels to capture load-dependent gas generation characteristics. These samples represent seven distinct transformer states: normal condition, low-temperature overheating, medium-temperature overheating, high-temperature overheating, partial discharge, low-energy discharge, and high-energy discharge. Initial defects are defined by concentrations marginally exceeding typical values, while critical conditions exhibit concentrations surpassing alarm limits by >200%, indicating imminent failure risk. Among the 1400 samples, 200 represent normal operating conditions. For the six fault categories, each containing 200 samples, the severity distribution encompasses the complete progression spectrum from incipient to critical stages. This classification scheme follows the IEC 60599 standard framework for transformer fault diagnosis.

The feature space consists of five key dissolved gas concentrations measured in parts per million (ppm): hydrogen (H_2_), methane (CH_4_), ethane (C_2_H_6_), ethylene (C_2_H_4_), and acetylene (C_2_H_2_). These gases are generated through thermal and electrical decomposition of insulating oil and cellulose materials under fault conditions.

### 3.2. Feature Engineering and Tensor Decomposition-Based Feature Selection

After expanding the features to 12 dimensions, Tucker decomposition is applied to select the most informative features from the extended 12-dimensional feature space, reducing dimensionality to 7 features while preserving critical fault discrimination capability.

**Figure 4:**
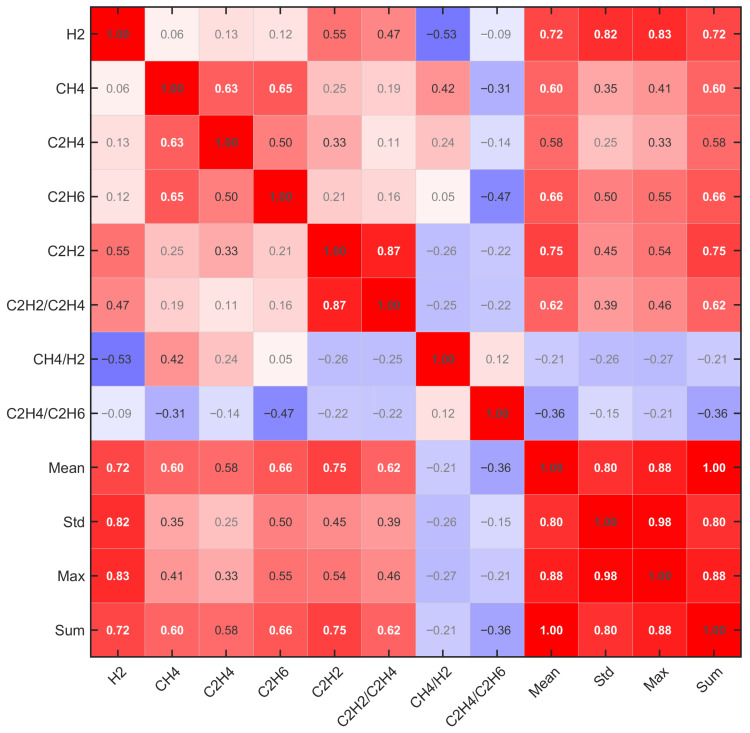
Feature correlation heatmap.

Figure 4 presents the correlation matrix among the 12 extended features. Strong positive correlations (red, >0.6) exist among original gas concentrations (H_2_, CH_4_, C_2_H_4_, C_2_H_6_, and C_2_H_2_) and statistical aggregates (Mean, Max, and Sum), indicating substantial redundancy. Gas ratios (C_2_H_2_/C_2_H_4_, CH4/H_2_, and C_2_H_4_/C_2_H_6_) show weak or negative correlations (blue regions) with absolute concentrations, suggesting complementary discriminative information. This correlation structure motivates feature selection to eliminate redundancy while retaining diverse fault signatures.

**Figure 5:**
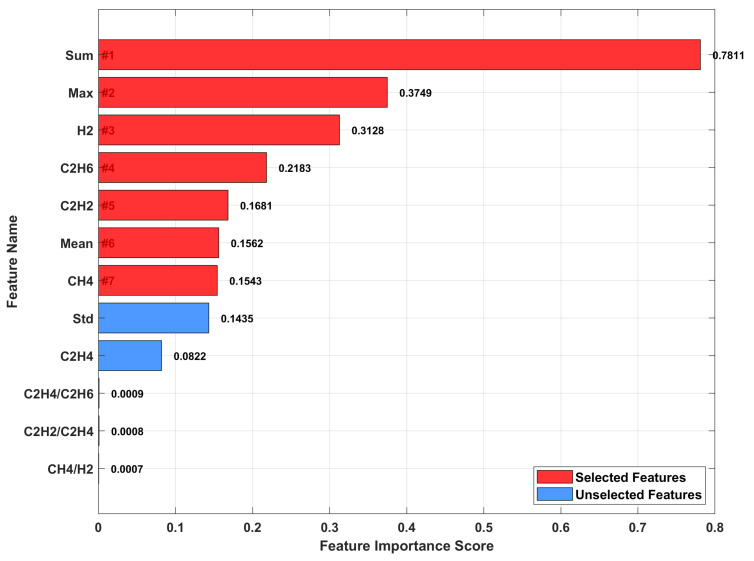
Feature importance score.

Figure 5 displays the feature importance ranking based on tensor decomposition. Seven features are selected (red bars): Sum (0.7811), Max (0.3749), H_2_ (0.3128), C_2_H_6_ (0.2183), C_2_H_2_ (0.1681), Mean (0.1562), and CH_4_ (0.1543). These features comprise both statistical descriptors and key gas concentrations, capturing the dominant fault patterns. The unselected features (blue bars) exhibit importance scores below 0.15, confirming their limited contribution. This selection achieves 41.7% dimensionality reduction while retaining 95% of discriminative information for fault classification.

### 3.3. Ablation Study on Feature Dimensionality

**Figure 6:**
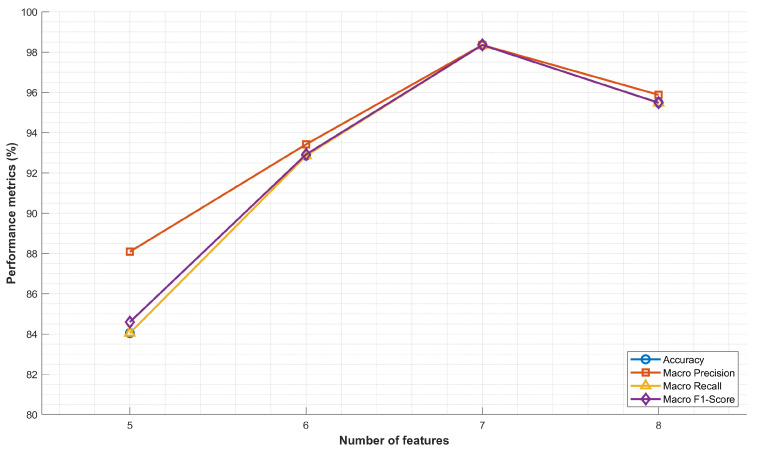
Optimal number of features.

This study evaluates the model’s performance by employing varying numbers of features to determine the optimal feature set size. As illustrated in Figure 6, feature subsets containing five, six, seven, and eight features were compared across four primary metrics: accuracy, Macro Precision, Macro Recall, and Macro F1-Score.

The experimental results demonstrate that the model achieved its peak performance when configured with seven features, attaining an accuracy of 98.35%, a Macro Precision of 98.35%, a Macro Recall of 98.35%, and a Macro F1-Score of 98.35%. This configuration consistently outperformed all other feature subsets across all evaluation metrics. Compared to the five-feature subset, the seven-feature configuration exhibited a substantial improvement of approximately 14.3% in accuracy and 13.9% in Macro F1-Score. In comparison to the six-feature subset, the performance gain was more moderate, with all metrics increasing by approximately 5.5%. Notably, when the feature set was expanded to eight features, the model’s performance slightly degraded compared to the optimal seven-feature configuration: accuracy decreased by 2.87%, and the Macro F1-Score decreased by 2.86%.

This performance trend indicates that while additional features initially contribute to the model’s discriminative capability, beyond a certain threshold, they may introduce noise or redundancy, thereby marginally compromising its generalization capability. The seven-feature set thus represents the optimal balance between model complexity and predictive performance, providing the most robust and accurate classification results while simultaneously maintaining computational efficiency.

### 3.4. Experimental Comparison

To establish a performance baseline, this study first evaluated two classic machine learning algorithms: K-Nearest Neighbors (KNN) and Decision Tree. Both models were trained and tested using the same seven features selected via tensor decomposition, thereby ensuring a comparison under controlled conditions where only the model architectures differed. For the KNN classifier, Euclidean distance and uniform weights were employed. Different numbers of neighbors (*k* = 3, 5, 7) were tested, and the optimal configuration was selected through three-fold cross-validation. The Decision Tree classifier was based on the CART algorithm, using Gini impurity as the splitting criterion. The maximum depth was limited to 10, and each leaf node was required to contain at least five samples to prevent overfitting.

Table 2 shows the performance of the simple baselines against the proposed method. The classical models exhibited clear limitations. The optimal KNN (k = 5) achieved an accuracy of 87.45%, with lower performance observed for k = 3 (86.12%) and k = 7 (86.89%). Decision Tree yielded the poorest performance, registering an accuracy of 84.23%. The 10.88 percentage point difference between the best-performing baseline and the proposed method highlights a fundamental architectural advantage. This disparity cannot be solely attributed to hyperparameter optimization, as the baselines were systematically optimized. Instead, it reveals that distance-based methods, such as KNN, and the axis-aligned partitions of Decision Trees are intrinsically unsuitable for modeling the complex, nonlinear relationships within the dissolved gas analysis (DGA) data, thereby confirming the need for our advanced architecture.

Having determined that simple classifiers are insufficient to capture the complexity of transformer fault patterns, this work advances by evaluating the proposed method against more sophisticated algorithms that incorporate advanced feature processing and learning mechanisms. To verify the effectiveness and superiority of the proposed tensor decomposition and multi-kernel SVM model based on the SSA, this paper selected several representative models as comparison objects, including KPCA-SSA-MKSVM and PCA-SSA-MKSVM as dimensionality reduction variants; a deep neural network (DNN) [26], a deep belief network (DBN) [27] with customized input features, an ensemble machine learning approach (Sklearn Classifier) [28] that incorporates application data and equipment-specific parameters, and a case-based reasoning method optimized by the k-optimal algorithm (K-OA-CBR) [29] were adopted for ratio feature extraction.

For the DNN approach, an MLP architecture with two hidden layers was employed, featuring 32 and 16 neurons, respectively. This structure utilized the ReLU activation function and a softmax output layer for multi-class classification. Network training was performed using the Adam optimizer with a learning rate of 0.001, a batch size of 32, and training for 100 epochs, incorporating an early stopping mechanism based on validation loss. The DBN model used the seven features selected by tensor decomposition as input to maintain consistency across all comparative methods. This network was configured with two hidden layers containing 50 and 25 units, respectively. It was trained using CD with a learning rate of 0.1 for 50 epochs of pre-training, followed by fine-tuning via backpropagation. Compared with the original DBN architecture that processes all available features, the customized DBN feature selection based on tensor decomposition in this study has the following advantages: 1. reducing the input dimension from 12 features to 7 features, thereby reducing model complexity and training time; 2. focus on features with proven discriminative ability quantified by core tensor contributions; and 3. reducing the curse of dimensionality in restricted Boltzmann machine layers. Despite its many advantages, the accuracy of the DBN is only 86.65%, which is 11.68% lower than that of the method proposed in this paper. For the Sklearn Classifier, the Logistic Regression model from the scikit-learn library was adopted. Its hyperparameters were set as solver = ‘lbfgs’, multi_class = ‘multinomial’, max_iter = 1000, C = 1.0, and random_state = 42 for reproducibility.

For the fairness of the experiment, all models were tested on the same dataset and evaluation metrics, and the parameter optimization of the models was carried out according to their respective algorithms. The experimental results are shown in the following chart.

**Figure 7:**
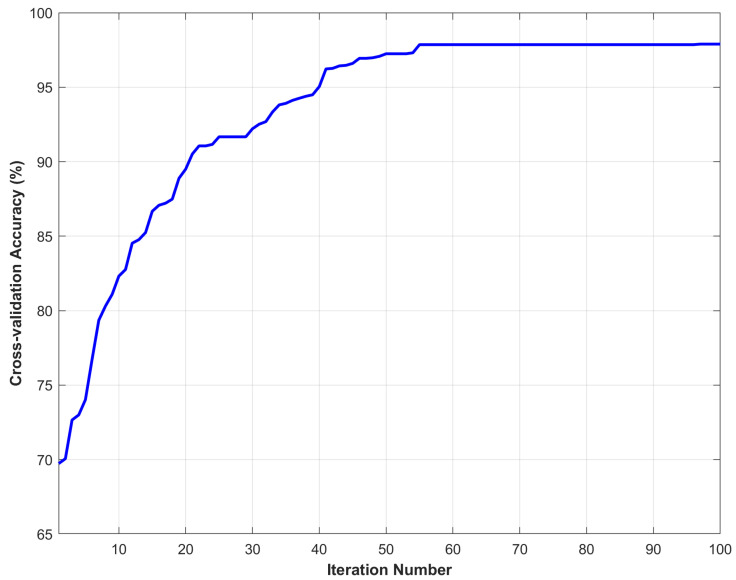
Cross-validation accuracy.

In this paper, a three-fold cross-validation strategy was employed. The dataset was randomly divided into three equal subsets. In each iteration, two subsets were used for training and the remaining one for testing. This process was repeated three times to ensure that each subset was used once as the test set. The following Figure 7 shows the iterative convergence curve of the model based on tensor decomposition and the multi-kernel SVM in terms of cross-validation accuracy. From the figure, it can be seen that in the first 10 iterations, the accuracy rate rapidly increased from about 70% to over 80%, indicating that the algorithm can find a better solution in the early stages. After 20 to 50 iterations, the accuracy rate continued to increase, gradually reaching over 95%. After 55 iterations, the curve tended to stabilize and eventually stabilized at around 98%. At this point, the algorithm had converged.

**Figure 8:**
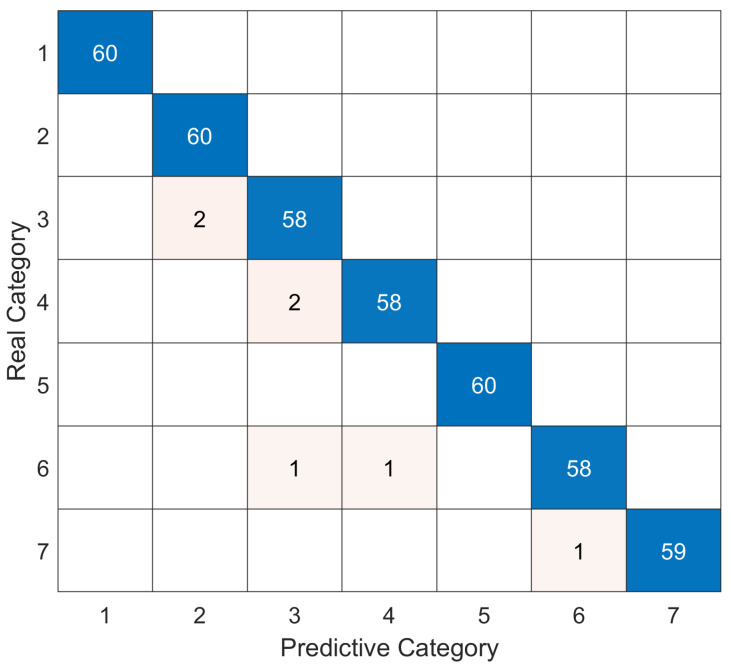
Confusion matrix on test set.

Figure 8 presents the confusion matrix of the proposed model on the test set, which demonstrates the classification performance across different fault categories. The matrix reveals that the model achieves high diagonal values with minimal misclassifications, indicating strong discriminative capability in distinguishing between fault types. The near-perfect diagonal pattern validates the model’s robust generalization ability and confirms its reliability for practical transformer fault diagnosis applications.

Table 3 summarizes the performance comparison among different diagnostic models. The proposed tensor decomposition and multi-kernel SVM model achieves the highest performance across all metrics, with an accuracy of 98.33%, precision of 98.50%, recall of 98.20%, and F1-score of 98.35%, substantially outperforming all baseline methods. Among the comparison models, KPCA-SSA-MKSVM demonstrates the second-best performance with 95.69% accuracy, which is 2.64 percentage points lower than that of the proposed method, indicating that nonlinear dimensionality reduction through kernel PCA offers advantages over linear approaches but still cannot match the comprehensive feature representation capability of tensor decomposition. The linear PCA variant (PCA-SSA-MKSVM) shows relatively lower performance at 92.87% accuracy, confirming that preserving nonlinear feature relationships is crucial for effective fault pattern recognition. The performance gap between KPCA-SSA-MKSVM and PCA-SSA-MKSVM (2.82 percentage points) further validates the importance of nonlinear feature extraction in transformer fault diagnosis. This study uses MLP for deep learning, but the results are only average, with an accuracy of only 92.33%, and the DBN reaches only 86.65% accuracy even with customized input features, suggesting that these methods may suffer from overfitting or insufficient feature representation in the context of transformer fault diagnosis with limited training samples. The Logistic Regression approach attains 93.71% accuracy by incorporating application-specific data and equipment parameters, demonstrating reasonable diagnostic capability but still showing a performance gap of approximately 4.62 percentage points compared to the proposed model. The K-OA-CBR method achieves 89.56% accuracy through optimized ratio feature extraction, yet its case-based reasoning mechanism appears less effective in capturing complex nonlinear fault patterns compared to the kernel-based classification strategy. The consistent superiority of the proposed method across all evaluation metrics, particularly the balanced performance in precision and recall, confirms its robust capability in minimizing both false positives and false negatives, thereby establishing it as a more reliable and practical solution for transformer fault diagnosis in power systems.

Furthermore, the proposed framework exhibits a total training complexity of O(T·P·K·n^2^·m) and requires approximately 132 s to train on a mid-range AMD Ryzen 5 CPU equipped with 32 GB of RAM. The Tucker decomposition feature selection phase requires 2 s; the primary computational cost is concentrated in the SSA optimization phase, which requires 120 s, which is 40% faster than grid search and converges more rapidly than PSO, the GA, and DE. The final SVM model training requires 10 s. The model is readily deployable, making it suitable for both industrial PC and server scenarios.

## 4. Conclusions

This study presents a comprehensive framework for transformer fault diagnosis that synergistically integrates Tucker decomposition-based feature selection with an MKSVM optimized by the SSA for DGA. The proposed method first constructs a three-dimensional tensor to encode the coupling relationships among samples, features, and fault categories, extracting seven optimal features from the original 12-dimensional feature space while preserving 95% of the discriminative information. Subsequently, the hierarchical search mechanism of the SSA enables joint optimization of kernel weights and hyperparameters, achieving globally optimal configuration of the multi-kernel SVM. Compared to benchmark algorithms requiring 78–92 iterations, the proposed framework demonstrates efficient convergence within 60 iterations. Experimental validation on DGA samples encompassing seven fault categories demonstrates superior performance, overall accuracy of 98.33%, balanced accuracy of 98.50%, recall of 98.20%, and F1-score of 98.35%, representing improvements of 2.64% to 11.68% over KPCA-SSA-MKSVM (95.69%), MLP (92.33%), and other baseline methods. Ablation studies further confirm that the selected seven features represent the optimal equilibrium point, as performance degradation occurs when features are reduced to six or increased to eight. The scalability and general applicability of this study are supported by two key points. First, the physicochemical principles governing fault gas generation remain universally consistent, regardless of whether the transformer capacity is in the kVA or MVA range. Second, traditional diagnostic methods, which rely on fixed empirical gas ratio thresholds, encounter scalability issues when applied to diverse oil–paper systems and transformer sizes. In contrast, the proposed Tucker decomposition employs a data-driven tensor analysis technique that automatically extracts discriminative feature combinations by capturing the latent multilinear structure within the DGA data. This ability to learn from data enables it to adapt to the more complex and nonlinear gas interaction patterns present in transformers of varying scales. These results establish the reliability of the proposed framework for transformer condition monitoring and demonstrate significant potential for enhancing power grid reliability through accurate early fault detection.

## Figures and Tables

**Table 1:** Optimization algorithm comparison for MKSVM hyperparameter tuning.

Algorithm	Best Accuracy	Mean Accuracy	Std Dev	Convergence Iter
PSO	97.82%	97.45%	0.28%	85
GA	97.65%	97.21%	0.35%	92
DE	97.91%	97.58%	0.24%	78
SSA	98.33%	98.15%	0.12%	60

Results averaged over 10 independent runs with population size = 30, maximum iterations = 100.

**Table 2:** Performance comparison between simple baseline models and the proposed method.

Model	ACC	Precision	Recall	F1-Score
KNN (k = 3)	86.12%	86.45%	85.80%	86.11%
KNN (k = 5)	87.45%	87.80%	87.10%	87.44%
KNN (k = 7)	86.89%	87.15%	86.60%	86.87%
Decision Tree	84.23%	84.50%	83.95%	84.22%
Proposed	98.33%	98.50%	98.20%	98.35%

All values are in percentage (%).

**Table 3:** Model performance comparison.

Model	ACC	Precision	Recall	F1-Score
KPCA-SSA-MKSVM	95.69%	96.00%	95.40%	95.70%
PCA-SSA-MKSVM	92.87%	93.20%	92.50%	92.85%
MLP	92.33%	92.60%	92.05%	92.32%
DBN	86.65%	87.00%	86.30%	86.65%
Logistic Regression	93.71%	93.80%	93.60%	93.70%
K-OA-CBR	89.56%	89.80%	89.30%	89.55%
Proposed	98.33%	98.50%	98.20%	98.35%

All values are in percentage (%).

## Data Availability

Data sharing not applicable.
